# Editorial

**Published:** 1870-09

**Authors:** 


### Editorial.

EXPLANATION.—The article in this number of the *Examiner*, on Vaccinal Syphilis, by W. B. Davis, M.D., of Cincinnati, appears to have been furnished to several periodicals simultaneously. We would have placed it under the head of *selected* articles, had we known what journal it should be credited to.

THE WOMAN'S HOSPITAL MEDICAL COLLEGE, OF CHICAGO.—An institution, with this title, has been organized in this city, for the special purpose of educating *females* for the practice of medicine.

It has a full faculty, composed of members of the profession, in good standing; and, we have reason to believe, prepared to give full courses of instruction in all the departments of medical science.

It is connected with the Woman's Hospital, 402 State Street, which has been in operation several years. The regular lecture term will commence on the first Tuesday in October next. We do not advise women to study or practice medicine as a profession; but, to such as are determined to do so, without our advice, we say, they will find the Woman's Hospital Medical College, of Chicago, worthy of their patronage.

For further information, see advertisement; or, apply to Prof. T. D. Fitch, Secretary of the Faculty.

---

Chicago Medical College, Medical Department of Northwestern University.—The new building, for this institution, is now complete. It is, in all respects, one of the best medical college buildings in the country, and its completion, in immediate connection with the Mercy Hospital, and the Davis Free Dispensary, gives to the Faculty all the means and facilities for as comprehensive a system of medical education as can be devised. The College, Hospital, and Dispensary are all in the same block; all new, and arranged with special reference to the highest comfort and convenience of the sick; together with the most perfect facilities for Clinical instruction; and all free from municipal, political, or any other outside influence or control. Every practical department, including Practical Medicine, Surgery, Obstetrics and Gyneæcology, Ophthalmology, etc., is provided with the facilities for Clinical instruction, in direct connection with the didactic courses in the College. The new building also contains an extensive museum and laboratory for instruction in Analytical and Practical Chemistry.

---

A GOOD CHANCE FOR A GOOD PHYSICIAN.

I wish to dispose of my house and two lots in the village of Two Rivers, with a good village and country practice. Said lots are conveniently and pleasantly located, and have a good house, barn, and other improvements. For particulars and reasons for wishing to sell, address C. C. CROCKER, M.D., Two Rivers, Manitowoc County, Wis.

### Mortality for the Month of July, 1870.

| | |
|---|---|
| Accident, burned in building | 1 |
| " burned by petroleum | 1 |
| " crushed | 1 |
| " explosion of gas generator | 1 |
| " narcotics, overdose of | 1 |
| " by team | 1 |
| " drowned | 15 |
| " by fall | 2 |
| " railroad | 3 |
| " scalded | 1 |
| Abortion | 1 |
| Anus imperforate | 1 |
| Anasarca | 1 |
| Apthæ | 1 |
| Apoplexy | 12 |
| Asthma | 1 |
| Atelectasis pulmonum | 1 |
| Aneurism of Aorta rupture of | 1 |
| Births, premature | 21 |
| Births still | 55 |
| Bowels, congestion | 1 |
| Brain, congestion | 16 |
| " covering, inflammation of | 1 |
| " disease of | 1 |
| " inflammation | 17 |
| " effusion on | 2 |
| " paralysis of | 1 |
| " softening | 1 |
| Bronchitis | 1 |
| " chronic | 1 |
| " capillary | 2 |
| Cancer of Breast | 1 |
| " abdomen | 1 |
| " kidneys | 1 |
| " paroted glands | 1 |
| " stomach | 7 |
| " " uterus | 1 |
| Childbirth | 1 |
| Chest malformation | 1 |
| Cholera Infantum | 354 |
| " morbus | 10 |
| Consumption | 41 |
| Convulsions | 99 |
| " puerperal | 1 |
| Croup | 5 |
| " diphtheretic | 1 |
| " membranous | 2 |
| Cyanosis | 2 |
| Cynanche tonsillaris | 1 |
| Cystitis chronic | 1 |
| " | 1 |
| Development deficient | 1 |
| Debility, general | 8 |
| Delirium Tremens | 2 |
| Diarrhœa | 85 |
| " chronic | 6 |
| " & complications | 7 |
| Diphtheria | 9 |
| Dropsy & apoplexy | 1 |
| Dysentery | 24 |
| " acute | 2 |
| Dyspepsia | 1 |
| Entero-colitis | 10 |
| " hepatitis | 1 |
| Enteritis | 24 |
| Erysipelas | 4 |
| Exhaustion from amputation of leg | 1 |
| Fever, congestive | 3 |
| " intermittent | 1 |
| " puerperal, | 9 |
| " remittent | 1 |
| " scarlet | 19 |
| " " complications | 1 |
| " " malignant | 1 |
| " typhoid | 14 |
| Gastritis | 3 |
| " chronic | 1 |
| Gastro-Enteritis | 2 |
| Hæmatemesis | 1 |
| Hemorrhage umbilical | 1 |
| Heart, congestion | 1 |
| " disease | 4 |
| " organic disease | 1 |
| " fatty degenerat'n | 1 |
| " valvular disease | 4 |
| Hemiplegia | 1 |
| Hydrocephalus | 14 |
| " acute | 7 |
| Intussusception | 1 |
| Inanition | 16 |
| Intemperance | 2 |
| " and apoplexy | 1 |
| Jaundice | 1 |
| Kidneys, Bright's disease | 1 |
| Liver, cirrhosis | 1 |
| " inflammation | 1 |
| Lungs, congestion | 3 |
| " emphysema | 1 |
| Malformation, general | 1 |
| Mouth-canker sore | 1 |
| Measles | 3 |
| " & complications | 1 |
| Meningitis | 11 |
| " cerebro-spinal | 2 |
| " tubercular | 4 |
| Old age | 12 |
| " and dropsy | 1 |
| Paralysis | 4 |
| " & inflammation of glands of the neck | 1 |
| Peritonitis | 4 |
| Paraplegia | 1 |
| Pleurisy | 2 |
| Pneumonia | 5 |
| " & complications | 1 |
| " typhoid | 2 |
| Pyæmia | 1 |
| Quinsy | 1 |
| Rheumatism | 1 |
| " inflammatory | 1 |
| Scrofula | 3 |
| Small-Pox | 2 |
| Spinal Cord, softening | 1 |
| Stomach, ulceration | 1 |
| Stomatitis | 2 |
| " gangrenous | 1 |
| Sunstroke | 21 |
| " & complications | 5 |
| Suicide by hanging | 1 |
| " " poison | 1 |
| Syphilis, hereditary | 1 |
| " tertiary | 1 |
| " | 1 |
| Tabes mesenterica | 40 |
| Teething | 15 |
| " & complications | 14 |
| Tetanus | 1 |
| " traumaticus | 2 |
| Tumor encephaloid, hemorrhage of | 1 |
| Urine suppression of | 1 |
| Varioloid | 2 |
| Uterus, hemorrhage of | 1 |
| Vitality deficient | 2 |
| Whooping-cough | 9 |
| " & complications | 4 |
| Total | 1118 |

### COMPARISON.

| | | |
|---|---|---|
| Deaths in July, 1870, 1118 | Deaths in July, 1869, 815 | Increase, 303 |
| Deaths in June, 1870, 720 | Increase, 398 | |

AGES.

| | | | | | |
|---|---|---|---|---|---|
| Under 1 | 578 | 10 to 20 | 23 | 70 to 80 | 14 |
| 1 to 2 | 186 | 20 to 30 | 50 | 80 to 90 | 5 |
| 2 to 3 | 35 | 30 to 40 | 73 | | 3 |
| 3 to 4 | 20 | 40 to 50 | 45 | | |
| 4 to 5 | 5 | 50 to 60 | 25 | Total, | 1118 |
| 5 to 10 | 28 | 60 to 70 | 28 | | |

| | | | | | |
|---|---|---|---|---|---|
| Males, | 594 | Females, | 524 | Total, | 1118 |
| Single, | 931 | Married | 187 | Total, | 1118 |
| White, | 1112 | Colored, | 6 | Total, | 1118 |

NATIVITY.

| | | | | | |
|---|---|---|---|---|---|
| Atlantic Ocean | 3 | England | 16 | Poland | 2 |
| Bohemia | 8 | France | 3 | Russia | 2 |
| Canada | 9 | Germany | 103 | Scotland, | 5 |
| Chicago, Native | 171 | Holland | 5 | Sweden | 11 |
| Chicago, Foreign | 594 | Ireland | 78 | Unknown | 7 |
| U. S., other parts | 75 | Isle of Man | 1 | | |
| Denmark | 1 | Norway | 24 | Total, | 1118 |

MORTALITY BY WARDS FOR THE MONTH.

| Wards. | Mortality. | Wards. | Mortality. |
|---|---|---|---|
| 1 | 6 | 20 | 31 |
| 2 | 31 | Accidents | 27 |
| 3 | 34 | County Hospital | 7 |
| 4 | 22 | Convent Good Shepherd | 1 |
| 5 | 24 | Canal Boat Olive Branch | 1 |
| 6 | 78 | Home for Friendless | 9 |
| 7 | 72 | Hospital Alexian Brothers | 2 |
| 8 | 99 | Immigrants | 2 |
| 9 | 103 | Jewish Hospital | 3 |
| 10 | 24 | Lake Hospital | 1 |
| 11 | 50 | Mariner's Hospital | 1 |
| 12 | 33 | Mercy Hospital | 3 |
| 13 | 18 | Protestant Orphan Asylum | 4 |
| 14 | 18 | St. Luke's Hospital | 1 |
| 15 | 118 | St. Joseph Orphan Asylum | 11 |
| 16 | 59 | Soldiers' Home | 1 |
| 17 | 90 | Suicide | 2 |
| 18 | 65 | | |
| 19 | 37 | Total, | 1118 |

THE SICK.—There are in the United States 1,360,000 "constantly sick," or twenty-four to each physician.

In the United States Army, one medical officer is provided to 333 men in regiments.

